# Method of estimating sea‐surface paleotemperatures through biotic proxies: A case study in Upper Paleozoic paleoclimatic, paleogeographic and paleotectonic reconstructions of Siberia

**DOI:** 10.1002/ece3.70265

**Published:** 2024-11-07

**Authors:** Vladimir I. Davydov, Eugeny V. Karasev, Elizaveta V. Popova, Vladislav I. Poletaev

**Affiliations:** ^1^ Department of Geosciences Boise State University Boise Idaho USA; ^2^ Borissiak Paleontological Institute of Russian Academy of Science Moscow Russia; ^3^ Kazan Federal University Kazan Tatarstan Russia; ^4^ Trofimuk Institute of Petroleum Geology and Geophysics of the Siberian Branch of the Russian Academy of Sciences Novosibirsk Russia; ^5^ Institute of Geological Science the National Academy of Science of Ukraine Kiev Ukraine

**Keywords:** database and tools, Late Paleozoic, paleobiota taxonomy, paleoclimate, paleotectonics, sea‐surface paleotemperatures, Siberian Platform

## Abstract

This study introduces a novel approach for quantitatively assessing sea‐surface paleotemperatures examined in the Upper Paleozoic of Siberia, utilizing the obtained in the region data as a case study of the use of this method. The method relies on evaluating the taxonomic composition and ecological proxies of biota. It utilizes a comprehensive dataset encompassing the geographic distribution and ecology of various biotic groups in Siberia and adjacent regions, leveraging the newly developed by the authors large PaleoSib database and partially the Paleobiology Database (paleobiology.org) The taxonomy has been used according to the database of Global Biodiversity Information Facility (gbif.org). Fossils collected from individual locations often exhibit a wide spectrum of paleotemperatures. To address this variability, we developed an algorithm for calculating average biotic paleotemperatures in each locality/time slice. Our computations of the available data have unveiled a coherent pattern of paleoclimate dynamics, particularly Sea Surface Temperature, across Siberian basins and surrounding areas during the Late Paleozoic era. These findings significantly contribute to a refined comprehension of paleoclimate and paleotectonic dynamics in the region during that specific time. To enhance paleotemperature analyses, we have integrated lithological indices with biotic ones, fortifying the overall methodology and furnishing a more robust framework for interpreting paleoclimate data. The method could be a helpful tool in regional and interregional studies, regardless of the utilized rock's age and fossil groups.

## Graphical abstract

This study introduces a novel approach for quantitatively assessing sea‐surface paleotemperatures examined in the Upper Paleozoic of Siberia. The method relies on evaluating the taxonomic composition of biota. It utilizes a comprehensive dataset encompassing the geographic distribution and ecology of various biotic groups in Siberia and adjacent regions, leveraging the newly developed PaleoSib database. An algorithm for calculating average biotic paleotemperatures in each locality/time slice has been developed utilizing the PaleoSib database. We aim to incorporate this method into the Paleobiology Database, enhancing its accessibility and fostering its adoption by the broader scientific community.

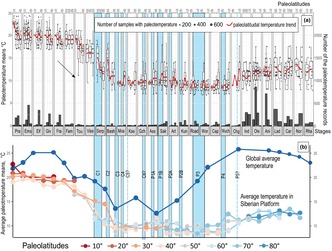

## 1 INTRODUCTION

The climate plays a pivotal role in shaping the Earth's system, directly influencing the development of its sedimentary stratosphere, hydrosphere, atmosphere, and biosphere. Therefore, investigating the dynamics of climate over time is crucial for gaining a comprehensive understanding of Earth's history, including the evolution of various fauna and flora groups, weathering processes, and the diverse compositions of sediments and minerals associated with sedimentary processes (such as coals, evaporites, laterites, etc.).

The primary methods used in reconstructing paleoclimates involve studying sediment composition and sedimentological indices that specified cold (e.g., dropstones, diamictites, glendonites), arid (calcarenites, evaporites, laterites), and humid climates (coals, peat) (Boucot et al., 2013b; National Research Council, 2006). In recent years, the determination of climate parameters has commonly relied on geochemical weathering indices (Baumgardner et al., 2014; Rasmussen et al., 2011). Additionally, oxygen and carbon isotopes, along with Ca/Mg ratios in biogenic carbonates, serve as indicators of oceanic water temperature (Berlin & Khabakov, 1960; Epstein et al., 1951; Lebrato et al., 2020; Veizer & Prokoph, 2015).

Advancements in modern mass spectrometers have significantly enhanced data accuracy on past Sea Surface Temperatures (SST) when studying biogenic phosphates (Joachimski et al., 2004). However, it is important to note that all geochemical methods for paleotemperature determination necessitate a sophisticated and costly instrumental base, and the processes are time‐consuming. For instance, SST data for the Late Paleozoic are currently available only for Southern China, where the climate, hydrography, physiography, and paleogeography exhibit distinct and specific regional characteristics.

A recently proposed method for modeling paleotemperatures involves estimating the atmospheric carbon dioxide (CO_2_) levels, as explored in various studies (Marcilly et al., 2022; Niezgodzki et al., 2017; Upchurch et al., 2015). Paleotemperatures are derived by positioning studied objects relative to climatic zones established through modeling based on atmospheric CO_2_ content (Roeckner et al., 2003). Carbon dioxide levels are assessed through the study of stable isotopes in paleosols and the stomatal index of plant leaves (Royer, 2001).

**FIGURE 1:**
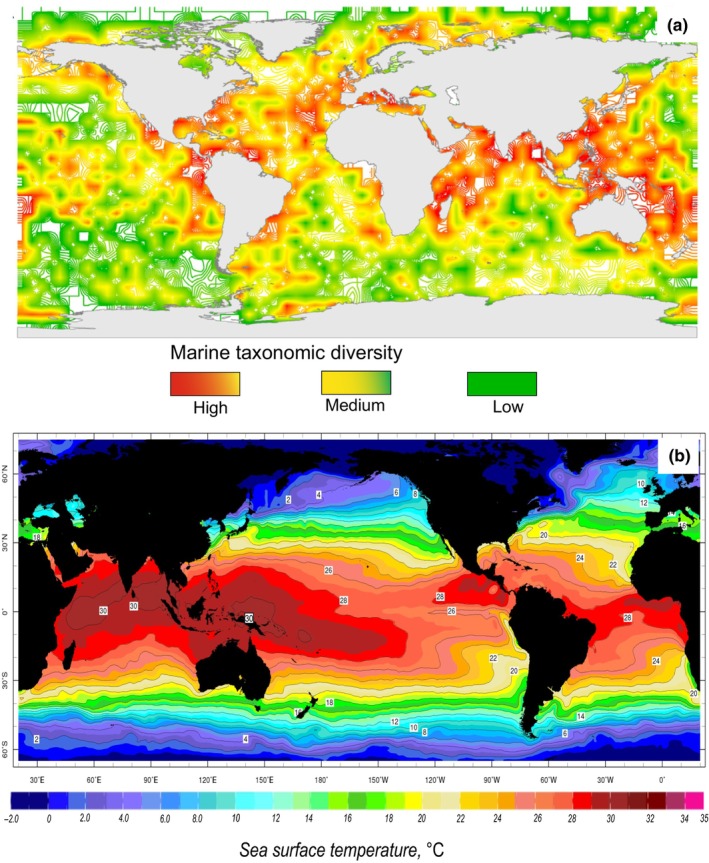
Maps of taxonomic diversity of species in the modern oceans (a) and average ocean surface temperatures. Taxonomic diversity is calculated as the number of species in 50 random samples in each 5° latitude‐longitude cell (51,670 species) (Costello & Chaudhary, 2017). The greatest biodiversity is observed in the tropical and subtropical zones. Diversity decreases toward the poles. (b) Distribution of mean annual modern ocean surface temperatures according to (Banzon et al., 2014). In the tropics and subtropics, as well as in the polar latitudes, the isotherms are at a considerable distance from each other and there are many taxa with a narrow thermal tolerance. At middle and high latitudes, isotherms are very concentrated, and taxa are adapted to exist within a wider thermal tolerance. At latitudes above 60°, tolerance decreases sharply (Bennett et al., 2021; Deutsch et al., 2020; Dorey et al., 2019; Stuart‐Smith et al., 2017; Sunday et al., 2011).

While lithological indices of climate are widely used in geology, a considerable amount of information on paleotemperatures, indicative of biotic living environments in diverse ecological settings, often eludes the broader geological community. Various flora and fauna groups alone can offer accurate insights into general climatic conditions in both terrestrial and marine environments (Figure 1).

**FIGURE 2:**
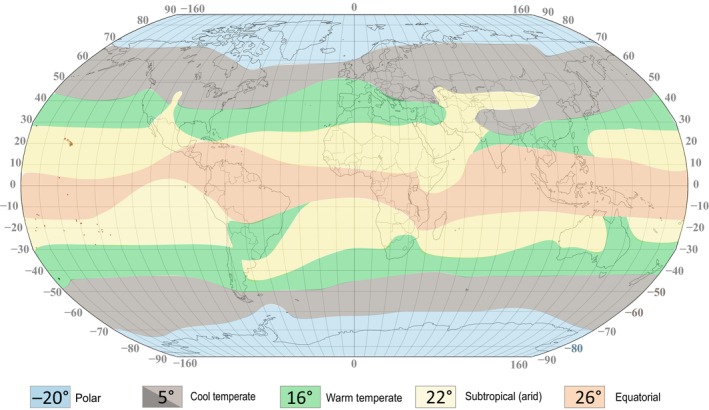
Simplified model of the modern climatic zones of the Köppen model (1923) in the present‐day oceans and average temperatures in each zone (map modified from Scotese et al. 2021). These zones shifted latitudinally through time, depending on the global climate fluctuations.

Paleontologists studying biotic groups across different climatic zones typically possess an understanding of general climatic parameters for specific taxonomic groups (e.g., corals, ammonoids, planktonic foraminifers, radiolarians). Nevertheless, sedimentological indices, such as gledonites and laterites, can sometimes accurately and quantitatively assess temperature ranges or environments. Surprisingly, biotic taxonomies are not consistently used for the quantitative evaluation of paleotemperatures. It is noteworthy that the renowned classification and map of modern climatic zones primarily rely on the distribution of terrestrial biota, particularly flora (Köppen, 1923; Köppen & Wegener, 1924; Kottek et al., 2006) (Figure 2). However, the paleontological community has not yet adequately responded to Köppen's attempts to utilize both biotic and sedimentological indices for developing Phanerozoic paleoclimatic models, providing only a generalized perspective on deep‐time climate.

Another method known as the “Coexistence Approach” (CA) has proven effective in quantitatively reconstructing terrestrial Tertiary paleoclimates, representing a plant‐centric methodology (Utescher et al., 2014). This approach involves considering the recent climatic distribution ranges of the nearest living relatives of each fossil taxon, without resorting to statistical processing or additional assumptions. Climatic requirements are derived directly from meteorological stations within the corresponding distribution area. However, recent observations have highlighted a potential drawback in the CA method, namely its susceptibility to statistical outliers and exotic elements due to the absence of a statistical framework (Grimm & Potts, 2016). Various theoretical and methodological concerns have been raised, with some suggesting discontinuing the use of the CA method and revisiting previous reconstructions using more robust methodologies (Grimm & Potts, 2016). To enhance the reliability of paleoclimate reconstructions, these authors advocate for developing (semi‐quantitative) methods that prioritize robustness over precision.

The paleotemperature estimation has been used with the GDGT (glycerol dialkyl glycerol tetraether) proxies method (Schouten et al., 2007). GDGTs are lipids synthesized by marine archaea, prevalent in both marine and terrestrial environments, and are a dominant prokaryotic group in the oceans throughout time (Howland, 2000). However, these biomarkers are thermally unstable and can be utilized only in well‐preserved Cretaceous and younger sediments. Moreover, their calibrations to Sea Surface Temperature are limited to approximately <28°C, rendering them unsuitable for recent tropical oceans or ancient hothouse climates (Tierney, 2012). The application of modern machine learning tools, such as OPTiMAL (Optimized Palaeothermometry from Tetraethers via MAchine Learning), holds the potential for improving temperature estimation and uncertainty representation by leveraging the relationship between ancient GDGT assemblage data and the structure of the modern calibration dataset (Jones et al., 2020).

We present here a prototype of a paleoclimate model developed using biotic paleotemperature proxies in Siberian Platform and the North‐East and South East of Russia during the Late Paleozoic. The focus of this study is on elucidating the methodology for determining paleotemperatures through biota, emphasizing primary trends in surface water temperature changes. The application of this method is demonstrated through an analysis of the Late Paleozoic paleoclimate in Siberia, considering the dynamics in connection with global and regional climate change and various levels of tectonic activity within the region. Notably, proposed here approach aligns with the principles used in determining temperatures from sedimentological indices, as utilized in the work of Boucot et al. (2013b), especially given the inclusion of taxa such as palms and crocodiles, which were previously used in their research.

This paper delves into the methodology used for the analysis of paleotemperature distribution over time and space, utilizing data from Siberia and surrounding areas. Leveraging the PaleoSib database, time‐slices datasets, and newly developed tools, we aim to recognize reliable biotic paleotemperature indices to gain a deeper understanding of the Late Paleozoic climate in Siberia.

## 2 MATERIALS AND METHODS

The PaleoSib database (https://zenodo.org/records/10553023) serves as the foundational element of our project and was principally compiled from the literature (see Supporting Information). The dataset encompasses information derived from stratigraphic correlation charts spanning various regions of Siberia and surrounding areas, covering the Devonian to the Triassic periods (a comprehensive list of references is provided in Appendix S1). In Russia, these charts are commonly utilized to consolidate sedimentological, biostratigraphic, and chronostratigraphic data from significant geological basins, including the Siberian Platform and its adjacent areas. The primary basin is further segmented into sub‐basins (or in Russian terminology, Structural‐Facial Zones), with stratigraphic successions categorized into chronostratigraphic units, at least at the stage level. The PaleoSib database includes all taxa we compiled from the literature and some of them could be with outdated taxonomy or even missing in the GBIF. All in all, we utilized only taxa with GBIF (Global Biodiversity Information Facility, https://www.gbif.org) numbers and robust taxonomy expected in this database.

For this project, we specifically focused on paleontological objects with reliable quantitative assessments of paleotemperature, such as ammonoids, marine bivalves, certain brachiopods, benthic foraminifera, nautiloids, and conodonts. Additionally, we included taxa whose temperature sensitivity in Siberia and surrounding areas distinctly changes with transitions from warm to cold climates, such as corals and fusulinids. To enhance the dataset, we incorporated some information from the Paleobiology Database (https://paleobiodb.org), primarily sourced from Permian–Triassic deposits in Siberia.

**FIGURE 3:**
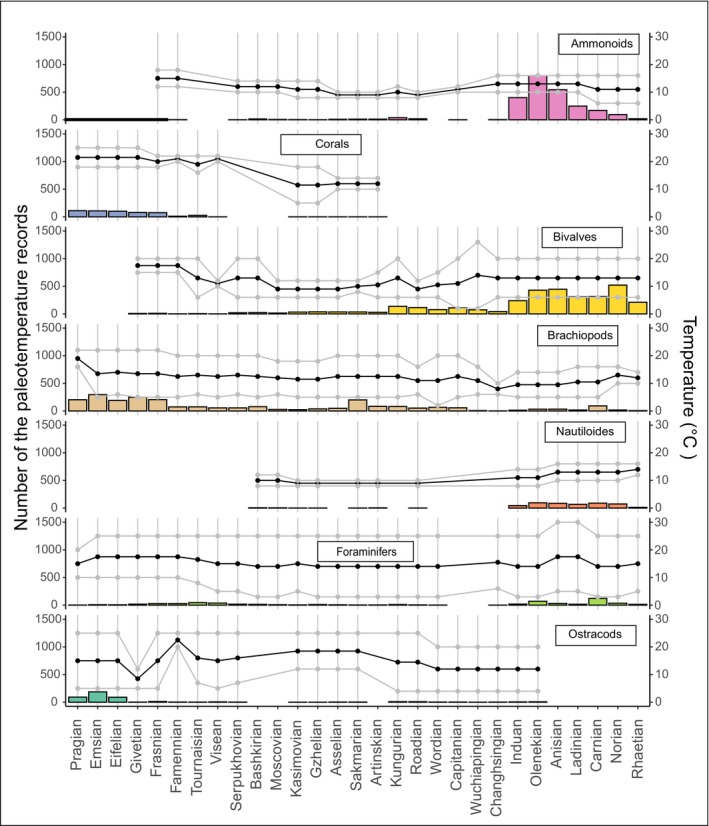
General trend of paleotemperatures and number of observations, general trend of paleotemperatures, and number of collections in the seven taxonomic groups in the PaleoSib database by age (Devonian‐Triassic). The number of collections is shown as a histogram, whereas the general trend of paleotemperatures—the means (solid line), minimal, and maximum values for the respective latitudes and climate zones. Gray lines indicate an error bar. During climatic warming fauna diversified and consequently the number of collections increases, whereas, during climatic cooling, the opposite processes are observed.

We systematically gathered and consolidated data from 170 published sources (S1), primarily originating from sub‐basins. The compilation encompasses specific locations mentioned in the literature. The PaleoSib database comprehensively spans all sub‐basins within the Upper Paleozoic and Triassic periods of the Siberian Platform, sub‐basins in the Western Siberia, North‐East, and South‐East Russia (within the Mongol‐Okhotsk suture). The number and dimensions of sub‐basins vary across different geological systems, contingent upon their designation in the correlation charts. Within the database, we have documented 23,580 stratigraphic records of taxa, each associated with assessed average paleotemperatures, originating from 5229 collections spanning the entirety of Siberia and Eastern Russia. Chronological subdivisions align with the stages acknowledged in the International Geological Time Scale (Gradstein et al., 2020). Figure 3 provides a statistical overview of biotic groups and the distribution of collections within the database.

## 3 TAXONOMIC LEVEL ASSESSMENT OF BIOTIC PALEOTEMPERATURES

The primary inquiry at the project's outset was: To what taxonomic level can paleotemperatures be accurately determined? While this question has been extensively addressed in the context of modern marine invertebrates (Heip et al., 1988; Warwick, 1993), it has received comparatively less attention in the study of ancient fossils. Few papers have delved into the variation of ecological and paleotemperature parameters within specific groups, and the outcomes regarding the influence of taxonomic level on climatic proxies remain contentious (Boyle et al., 2008; Heiri & Lotter, 2010; Lane, 2007; Punyasena, 2008). While some studies advocate for species as the best indicators of local climate, others defend family‐based climatic proxies (Punyasena, 2008).

**FIGURE 4:**
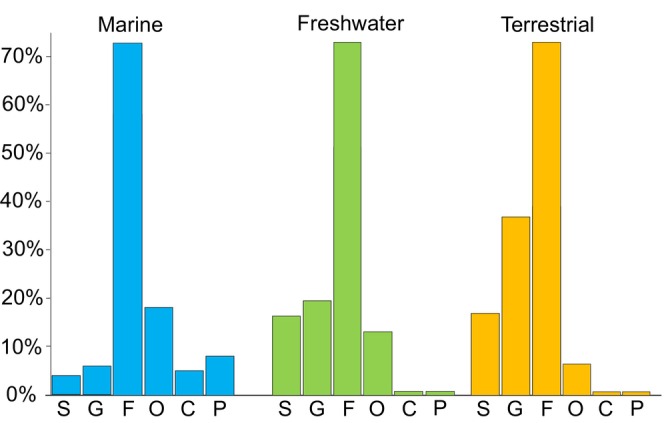
The percentage of taxa at different taxonomic levels for marine, freshwater, and terrestrial community types, and the proportion of taxa at different taxonomic levels sufficient to define ecological patterns (including temperature) in general terms; C, class; G, genus; F, family; O, order; P, phylum; S, species (After Bevilacqua et al., 2021). The figure shows that for reliable estimates of ecological conditions for marine communities over large areas, it is best to use data at the level of families and, to a lesser extent, orders; for freshwater and terrestrial communities—at the level of families, genera, and species.

We recognize that taxa at different taxonomic levels, such as species, genus, and family, often occupy similar ecological niches due to shared evolutionary histories and ecological responses under certain conditions (Clarke & Warwick, 1998). Consequently, higher taxonomic levels can be used to quantify ecological communities, a concept known as taxonomic sufficiency (Ellis, 1985). Recent studies on taxonomic sufficiency in modern ecological communities have examined various large ecological units, spanning marine, freshwater, and terrestrial habitats (Bevilacqua et al., 2021). The results suggest that, regardless of habitat type, identification at the genus or family level is sufficient to detect changes in communities responding to natural sources of ecological variability (Figure 4). Species‐level analysis is deemed necessary primarily for local and regional studies (Figure 4) (Jones, 2008; Olsgard & Somerfield, 2000; Terlizzi et al., 2014). Additionally, it is crucial to note that taxa of higher rank, such as families, exhibit less precision in determining general ecological parameters and may significantly differ from parameters established by species and genera within their distribution areas (Figure 4) (Carranza et al., 2011; Losos, 2008).

## 4 ESTIMATES OF PALEOTEMPERATURES OF TAXA

In our project, the assessment of paleotemperatures adhered to the following principles. Leveraging modern and updated data for various biotic groups, often at the family or order level (primarily for the Triassic and younger periods), facilitated the accurate estimation of habitat ranges for specific taxa across different taxonomic levels (Bevilacqua et al., 2021). Several families of ostracods and bivalves went from Devonian through the Permian–Triassic event, although their diversity at the event reduced drastically (Crasquin & Forel, 2014; Forel & Crasquin, 2022; Huber, 2015), and their paleotemperature evaluation at the family level during Late Paleozoic and Triassic is consistent. The ecological and paleoenvironmental data from the regional studies in Siberia and surrounding areas (Abramov, 1970; Abramov & Grigorieva, 1983; Abushik, 1990; Biakov, 2010, 2012, 2020; Bushmina & Kononova, 1981; Bychkov et al., 1976; Kurushin, 1992; Kutygin et al., 2020, 2024; Nikolaeva & Neustrueva, 1999; Polenova, 1960; Sarycheva, 1977; Sarycheva et al., 1963; Sobolev, 1989), were also analyzed in respect of the bathymetry and paleotemperature evaluation. The entire fossil assemblage from each record and the taxa specific to paleotemperature index‐value, i.e., warm‐water (corals, reefal facies) and cold‐water (agglutinated foraminifera), were considered in the paleotemperature evaluation procedure. The paleotemperature assessment of foraminifera and bivalves was completed using the expert's knowledge (Biakov, 2020; Davydov, 2014, 2016).

**FIGURE 5:**
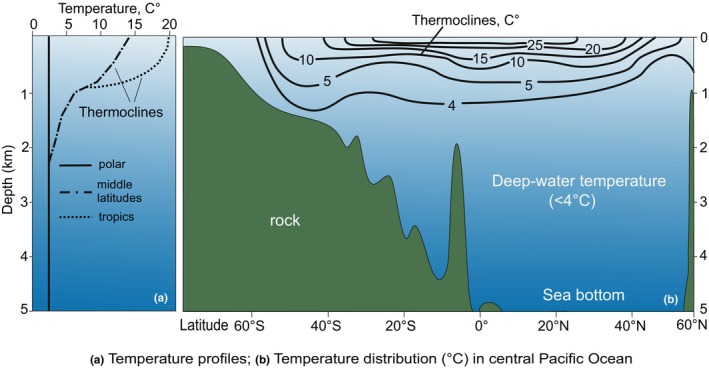
Present‐day temperature distribution in the central part of the Pacific Ocean. (a) The vertical temperature cross‐section at depths. The thermocline between warm and cold water is located at a depth of ~700 m; (b) the longitudinal cross‐section of the distribution of temperature isotherms over the latitudes. Most ocean water is colder than 4°C (modified from Pinet, 2019, p. 560).

We considered the latitudinal biodiversity gradient, recognizing that biotic diversity tends to be higher in warm‐water environments compared to cold water (Davydov, 2014; Pianka, 1966; Willig et al., 2003). The elevation of biodiversity in similar facies and bathymetric settings across different paleolatitudes indicates basin water warming and vice versa (Figure 3). We considered also the facies, environments, and bathymetry of the biotic groups. Shallow‐water environments were evaluated to realistically reflect the temperatures of surface waters, whereas transitional deep‐water settings exhibit permanent cold or intermediate cool temperatures. For the analysis of paleotemperatures in Siberia, the assessment focused solely on groups occurring in shallow water at depths less than 250–300 m. Consequently, shallow‐water faunas were indicative of higher temperatures compared to those of deeper water. This criterion applied universally to all benthic organisms, are extended into some nektonic faunas (e.g., ammonoids, and conodonts). Therefore, for the analysis of paleotemperatures in Siberia, shallow‐water benthic groups and nektonic ammonoids and conodonts as they appear to have inhabited the upper 250 m of the water column (Sessa et al., 2014; Sweet, 1988), thriving in shallow water conditions were considered (Figure 5).

The evaluation of paleotemperatures in Siberia involved several parameters. The position of taxa considered relative to climatic zones: (1) equatorial, (2) subtropics, (3) warm temperate, (4) cool temperate, and (5) polar (Boucot et al., 2013a) (For instance, the presence of migrants from a warm‐water climatic zone (e.g., corals among cold‐water faunas) in the studied assemblages characteristic of high latitudes provided grounds to associate that stratigraphic level in a given location with warmer, albeit short‐term, conditions.) Conversely, the presence of cold‐water conditions indices in assemblages of warm‐water taxa might indicate potential trends toward cooling. Furthermore, we incorporated the following methodological approach. Initially, taxa characteristics specific to warm‐water and cold‐water habitats were distinguished. All deep‐sea taxa residing at depths exceeding 250–300 m were classified as cold‐water species (Figure 5) (Pinet, 2019). Warm‐water taxa were identified as those dwelling at depths of 250–300 m or shallower in tropical and subtropical zones. In other climatic zones, they were found at much shallower depths (Figure 5). For cold‐water taxa, the search query “taxon name” + cold‐water was utilized in internet online searches, followed by an analysis of the publications, where certain taxon has been found and obtained from the web search results. Taxa exclusively found in deep‐sea conditions (9% of the analyzed collections) were excluded from the analysis, as their occurrences do not directly reflect Sea Surface Temperature (SST).

Due to the dynamic changes in global and regional climate conditions over time, taxa undergo multidirectional migrations in response to these alterations. For instance, during periods of global warming, warm‐water taxa may be found in middle and even higher latitudes alongside cold‐water forms (Figure 5), as the latter generally exhibit better adaptability to warming environments (Bennett et al., 2021; Dorey et al., 2019). To address this, we provisionally excluded overlapping taxa from consideration (~3% of the analyzed species), as these rare exotic species had minimal impact on establishing average temperatures for specific geographic areas and chronostratigraphic times. Evaluating such taxa requires individualized scrutiny in each particular case, demanding substantial time and specialized knowledge.

Shallow‐water species in tropical zones (≤300 m depth) exhibit average habitat temperatures ranging from approximately 20 to 35°C, whereas those in subtropical regions range from 18 to 25°C. In middle latitudes, temperatures range from 12 to 20°C, and in high latitudes, they range from 4 to 10°C, with polar regions experiencing temperatures from 0 to 4°C (see Figures 1b and 2b) (Pinet, 2019; Segar & Segar, 2018). Under deep‐water conditions below 250–300 m, but up to around 500 m, habitat temperatures in the tropics fall below ~10–12°C, in mid‐latitudes they range from 5 to 7°C, and in high latitudes above 60° (N–S), temperatures vary by about 1°–4° even in shallow water (Figures 1b and 5) (Miller & Wheeler, 2012). Additionally, these factors are influenced by climatic seasons, sea‐ocean currents, and local and regional settings (such as the presence of large rivers, mountain ranges with glaciers, anomalous salinity of deep‐water depressions, underwater volcanism, etc.) (Pinet, 2019; Segar & Segar, 2018). It is essential to note that these parameters were not considered in our project.

## 5 THE ALGORITHM FOR CALCULATING AVERAGE BIOTIC PALEOTEMPERATURES

Considering substantial temperature variations (temperature gradient) within the same taxon (Bennett et al., 2021; Dorey et al., 2019; Goldstein & DellaSala, 2020; Nati et al., 2021; Williams et al., 1997–2007), the temperature distribution of different taxa may significantly overlap not only within a stratigraphic unit and region but also within a single locality (Figure 4). To address this issue and establish temperatures approximating existing values, we developed an algorithm to calculate the average biotic temperature in the 29 stadial time slices (Devonian‐Triassic). The algorithm utilized Tukey (1977) method to eliminate the outliers. This is used because of the nature of the data (non‐parametric distribution). The obtained results then analyzed with Hodges‐Lehman to estimate median that is highly robust to deviations from normality.

**FIGURE 6:**
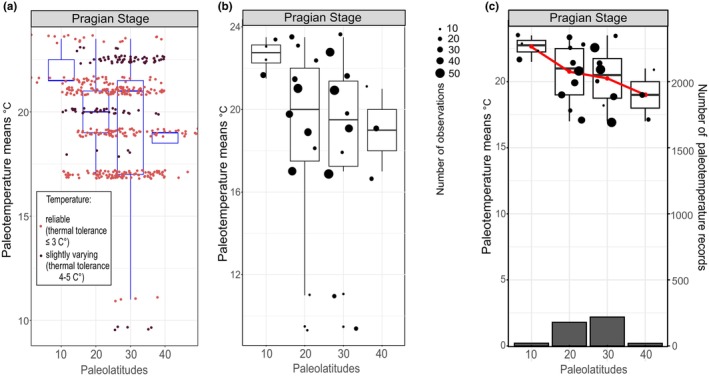
The sequence of data filtering with statistical methods to obtain the Pragian data analyzed the dynamics of the paleotemperatures over time in Siberia. See the explanations in the text; (a) Visualization of the obtained values for the average biotic paleotemperatures in different paleolatitudes; (b) Visualization of the obtained values of identical temperature variations; the size of the dots shows the number of records of the corresponding interval; (c) the final result of temperature estimation for each paleolatitude; the red line shows the trend of paleotemperature change depending on paleolatitude obtained with Hodges‐Lehman statistics (Hodges & Lehmann, 1963). The bar charts at the bottom—the quantity of the data.

The average biotic paleotemperatures for paleolatitudes in Siberia and surrounding areas were determined using a 10‐degree palaeolatitudinal bin for all 29 studied stages. Here we describe the algorithm for obtaining average temperatures, using the Pragian Stage as an example (Figure 6, Table 1). First, data from the Pragian Stage in the database (DB) is compiled, and only taxa with an average thermal temperature tolerance of <5°C are selected. This results in 448 records of taxa found in the studied sections (Figure 6). Of the 448 records, 347 taxa demonstrated narrow temperatures (variations ≤3°C) and 101 taxa with slightly varying average temperatures (variations 3–5°C). The average between the maximum and minimum temperatures is calculated for each record. All taxa are grouped according to paleolatitudes with a 10° bin. The resulting data can be visualized in the box plots (Figure 6a). Taxa with identical temperature variations are then grouped. To improve sampling reliability, outliers are excluded using the Tukey method (Tukey, 1977) (Figure 6b). Next, the main statistical characteristics (Table 1) are obtained using the non‐parametric Hodges‐Lehman estimate (Hodges & Lehmann, 1963; Wilcox, 2021), yields, and average temperature value for each paleolatitude since our data on temperature variations have an abnormal distribution. The final temperature estimate for each paleolatitude is shown in Figure 6c, where the red line shows the trend of seawater temperature variation depending on the latitudinal position of collection within Siberia.

**TABLE 1:** Main statistical characteristics of taxa for paleolatitudes in the Pragian stage.

Stage	Paleolatitude	*n*	*n*.int	HLM	Q1	Q3	IQR
Pragian	10	21	4	22.625	22.3	23.1	0.88
Pragian	20	181	11	19.625	17.5	22.0	4.50
Pragian	30	226	10	19	17.3	21.4	4.13
Pragian	40	20	3	19	18.0	20.0	2.00

*Note*: Columns in the table: *N* is the number of records on temperature variations of taxa; *n*.int is the number of unique temperature intervals; HLM is the value of the temperature estimate for the corresponding paleolatitude using the Hodges‐Lehman metric; Q1, Q3 are the first and third quartiles; IQR is the interquartile range.

## 6 PALEOSIB DB VISUALIZATION TOOLS

**FIGURE 7:**
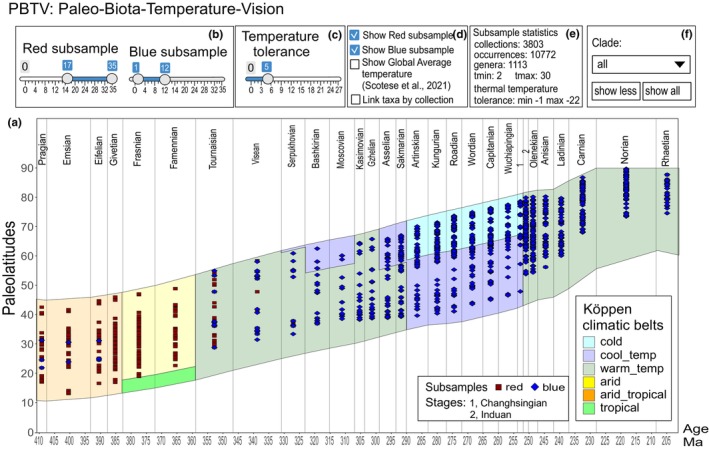
Interactive graphic of the distribution of the position of taxa with given paleotemperatures in time and space in the Siberian Platform during the Upper Paleozoic (https://mironcat.shinyapps.io/pbtv/). The color in the center shows the climatic zones according to Boucot et al. (2013b) divided into tiers by vertical lines. Other explanations are in the text.

To enhance accessibility and facilitate data analysis from PaleoSib, a dedicated visualization tool has been developed, offering a comprehensive view of taxa paleotemperature distributions relative to paleolatitude within the chronostratigraphic framework (refer to Figure 7). The core analytical component of this tool is the PBTV service (Paleo‐Biota‐Temperature‐Vision), a web application crafted in the R language and based on the Shiny Apps web service (https://mironcat.shinyapps.io/pbtv).

The primary window of the service presents an interactive chart illustrating the distribution of all established biota temperatures relative to paleolatitude (see Figure 7a). The latitudes for the Siberian Platform, Western Siberia, and Eastern Russia are depicted according to the plate tectonic model from the PaleoAtlas for GPlates and the PaleoData Plotter Program (Scotese, 2015). PBTV allows the creation of two independent sample sets (red and blue) using temperature ranges that align with the selected criteria. These scales facilitate the selection and display of warm‐water (red grades) and cold‐water (blue grades) taxa within the PBTV service (see Figure 7b). These sets are conditional, developed for convenience, and either or both grades can be used. These grades cannot be named as cold or warm, because the temperature range in each grade is dynamic and can be adjusted from cold to warm temperatures. The range of the temperature for each grade can be set by users according to their goals and wishes. An additional scale to the right (see Figure 7c) enables setting the boundaries of thermal tolerance within the ample, essentially limiting the width of the temperature range in which a particular taxon can exist.

On the chart, red and blue squares represent the position of corresponding taxa with certain paleotemperature. Two checkboxes (see Figure 7d) provide control over the display of the red and blue samples, allowing for a clear view of overlapping, dominating, or absent taxa samples. The overlap area is particularly crucial for validating established temperatures, as it aids in identifying taxa with conflicting temperature ranges. This process facilitates resolving discrepancies and determining whether specific taxa exhibit a wider thermal tolerance than conventionally considered. Two additional checkboxes (see Figure 7d) enable the visualization of global temperatures established using lithologic indices and geochemical proxy results (Scotese et al., 2021) and the presence of collections across different parts of the chronological scale. The subsequent block (see Figure 7e) displays key statistics of the sample, including the number of selected records in the database, taxa, collections, and temperature range.

**FIGURE 8:**
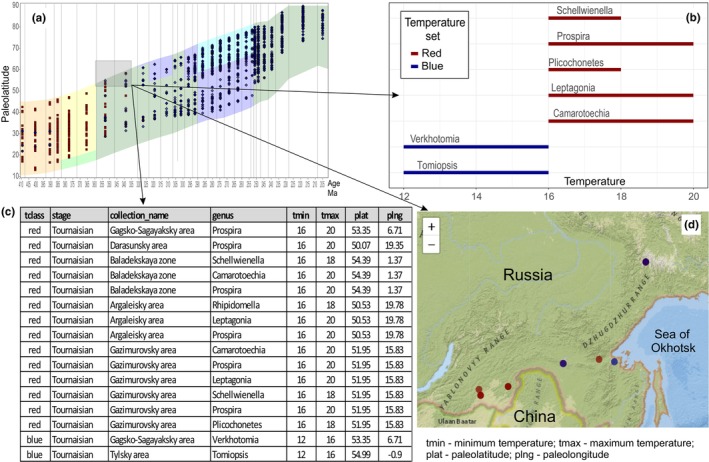
Visualization tool for paleotemperature and biotic data in the Siberian Platform and surrounding areas through time. (a) The distribution of temperatures relative to paleolatitude on the Siberian Platform, and North‐East and South‐East Russia (within the Mongol‐Okhotsk suture area). The paleolatitude data were obtained from the GPlates source (Boucot et al., 2013b). Each point on the graph represents a temperature from a specific collection in a particular sub‐basin; (b) the second window with the list of all taxa at the selected point, and their thermal tolerance; (c) a list of collections from different sub‐basins; (d) the position of the selected collections on a modern geographical map.

**FIGURE 9:**
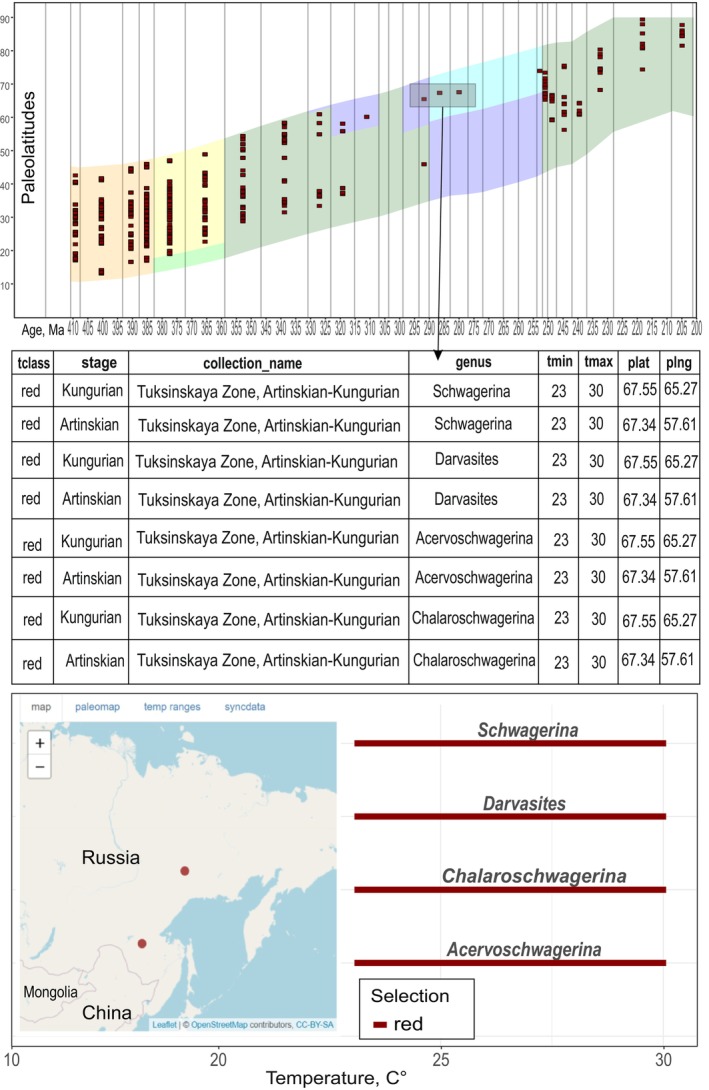
The PBTV service provides the immediate visualization of temperature deviations from the overall trend. In this case, it is shown that in the Tuksynskaya sub‐basin, paleotemperatures are characteristic of a tropical belt. This means that during the Permian period, this sub‐basin was located far to the south of the Siberian Platform and other terrains with cold‐water fauna. The square and arrows indicate the position of the Tuksynskaya sub‐basin biota, the location of this zone on the map, and the range of paleotemperatures for each taxon.

The PBTV service also allows for interactive selection of specific points on the graph (see Figure 8a), with their positions immediately displayed on the map below the distribution graph of taxa over the timescale (see Figure 8c,d). On the left side of the map (see Figure 8b,c), an automatically generated list of all selected taxa with their main attributes is presented. These attributes include their association with the red or blue group of taxa, collection age, absolute age in millions of years, subbasin (sub‐basin) name, full stratigraphic distribution, taxon's Latin name, mean temperature of its habitat (min.‐max.), modern and paleo‐coordinates, and the temperature range of each highlighted taxon (see Figures 8 and 9). Clicking on any taxon or group of taxa provides taxonomy and paleotemperature indices, along with their real positions on the map (blue and red dots) (see Figure 8b–d). These capabilities collectively aid in assessing issues related to conflicting paleotemperatures and paleotectonics. If necessary, the certain version of available paleogeographic map projection can be selected to show the selected points in paleo‐projection.

An illustrative example from the southeastern periphery of the Siberian Platform and South‐East Russia highlights the tool's utility. The Tukhinsky sub‐basin, distinguished by Ruzhentsev and Nekrasov (2009), presents a case where existing geodynamic models are contradictory (Khanchuk et al., 2015; Parfenov et al., 2001; Safonova & Santosh, 2014). While Scotese's paleogeographic map places it within the Siberian Platform terrains with the cool‐water setting during the Permian period, whereas the encountered taxa suggest a tropical zone with mean temperatures around 20–22°C (see Figure 9). This example underscores how the PaleoSib database project, when fully implemented, can practically contribute to resolving paleotectonic problems for geologists across different specialties, without requiring extensive knowledge of paleontology, taxonomy, and ecology. The tool relies on basic information such as names, locations, and bathymetry.

## 7 BIOTIC PALEOTEMPERATURE INDICES AND THE POSITION OF THE SIBERIAN PLATFORM IN TIME AND SPACE DURING THE UPPER PALEOZOIC AND LOWER‐MIDDLE TRIASSIC

The estimations of average biota temperatures offer insights into the prevailing climate trends in Siberia throughout the Late Paleozoic era. Two primary factors influencing paleotemperature variations in the region are paleotectonics and global‐regional paleoclimate fluctuations. The lateral movement of large plates, especially the expansive Siberian Platform, may lead to traversing multiple climatic zones, causing changes in paleotemperatures within shallow water basins on the platform. Additionally, the boundaries of climatic zones globally shift toward the poles during periods of global warming and toward the equator during global cooling (Brito‐Morales et al., 2020; Burrows et al., 2011).

The Late Paleozoic epoch is marked by a distinctive shift in global climate, often termed the “Late Paleozoic Ice Age (LPIA)” (Fielding et al., 2008). Despite this, glaciations occurred intermittently, and the climate during interglacial periods could be relatively warm (Davydov, 2014; Davydov et al., 2013; Fielding et al., 2023; Montañez, 2022; Montanez & Poulsen, 2013). In interpreting paleotemperatures, both factors—tectonics and global climate—must be considered, especially for the Siberian Platform and surrounding areas, where the significant influence of both elements is evident (Boucot et al., 2013b).

According to paleomagnetic data, during the early Devonian, the southern part of the Siberian Platform occupied paleolatitudes ranging from 20° N (modern northern part) to approximately 40° N in the north (modern southeastern part) (Domeier & Torsvik, 2014; Metelkin et al., 2015). The average paleotemperature in the region during this period varied between 22 and 17°C, contingent on the latitudinal location of the data on the platform (paleo‐north–paleo‐south). The Siberian Platform, during this time, was generally situated in the subtropics (~20–25° N), with its northern margin possibly extending into the warm‐temperate zone (Figure 2). From the Eifelian to the Frasnian, a marginal average temperature drops of about 1–2°C occurred, likely associated with a slight northward migration of the Siberian Platform. This supposition aligns with the simultaneous temperature drop on the platform and the significant global warming trend at that time (Joachimski et al., 2004; Scotese et al., 2021). This trend of declining paleotemperatures persisted until the end of the Devonian and the onset of the Carboniferous, with a minor overall decrease in temperatures on the Siberian Platform.

**FIGURE 10:**
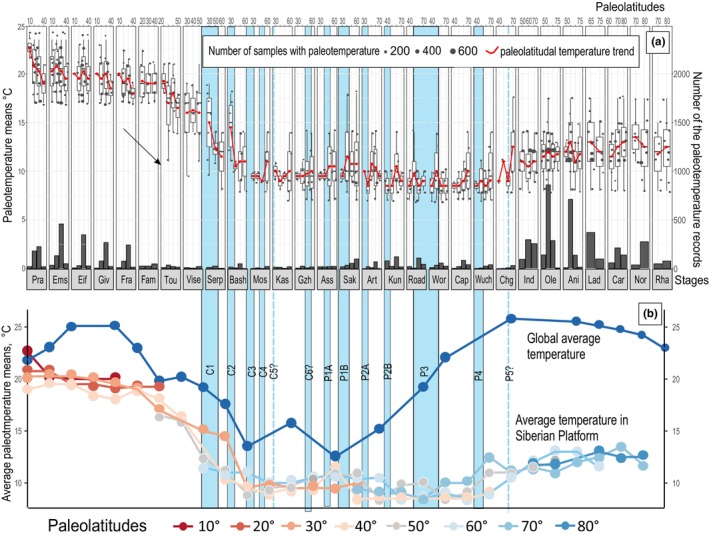
Distribution of paleotemperatures on the Siberian Platform during the Devonian‐Triassic. (a) Mean biotic paleotemperatures and their distribution relative to the latitudinal position on the Siberian Platform. Dots show the value of the thermal tolerance of taxa. Below—is the occurrence of all taxa in different sub‐basins and separate outcrops. Blue rectangles intersecting both figures are glacial episodes in Gondwana and Siberia. The arrow on the left in (a) shows the moment of the sharp tectonic displacement of the SP toward the north. (b) Trends of average paleotemperatures on the Siberian Platform relative to the latitudes of the Siberian Platform proposed by (Boucot et al., 2013b), and their comparison with global average paleotemperatures from (Scotese et al. 2021).

During the Visean period, a substantial decline in paleotemperatures by ~3–5°C occurred (Figure 10a). Concurrently, significant warming and extensive reef development characterized the global Visean climate. These observations suggest that the speed of the Siberian Platform's northward movement approximately doubled at the beginning of the Visean (Figure 10).

The Serpukhovian‐Bashkirian interval witnessed a drastic drop in average temperatures in the Siberian Platform basins by ~7–8°C, associated with documented global glacial episodes (Figure 10) (Fielding et al., 2023; Griffis et al., 2023; Rosa & Isbell, 2021). Temperature trends during this period show a clear distinction between the paleo‐north and paleo‐south of the platform (Figure 10a), indicating the platform's location in the middle latitudes (~45°–50°). Glacial deposits were unlikely to form in this region during this time.

**FIGURE 11:**
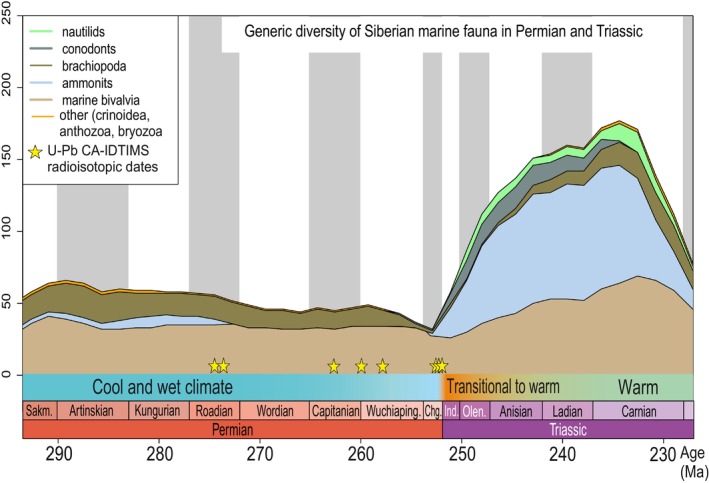
Generic diversity of Siberian marine fauna in the Permian and Triassic (Davydov, 2021). The climate remained almost unchanged during the Permian, while a sharp warming occurred in the Triassic, which led to an increase in diversity.

From the middle of Pennsylvania (Moscovian) through the end of the Middle Permian, spanning approximately 37 million years, paleotemperatures in the Siberian Platform basins exhibited slight variations, ranging from 4 to 10°C (Figure 10). Temperatures at all latitudes in the Siberian Platform showed minimal differences, undergoing slight decreases during proposed glacial episodes (P1–P3) and slight increases during documented interglacial times in the Southern Hemisphere (Figure 10). This suggests that the Siberian Platform maintained a relatively stable position at approximately the same latitudes, possibly slowly migrating northward. Biodiversity during this period remained unchanged (Figure 11).

**FIGURE 12:**
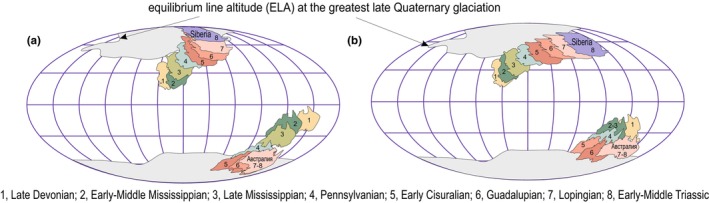
Paleogeographic reconstructions of the position of the Siberian and Australian cratons within climatic zones during the Late Paleozoic. 1—According to paleomagnetic and data on detrital zircons; 2—estimated from paleotemperature biotic data from PaleoSib database.

Despite being in middle latitudes, the Siberian Platform displayed the unexpected discovery of glacial‐marine deposits in the Middle Permian, primarily in its northernmost region (Figure 12 a, b). These deposits, found at three stratigraphic levels (late Roadian‐early Wordian; early Wuchiapingian; (?) middle Changhsingian), indicate a relatively constant paleotemperature climate. Toward the end of the Changhsingian and during the Induan, a notable increase in average paleotemperatures from ~5 to 12–13°C occurred (Figure 10b). Throughout the Triassic period, average paleotemperatures remained around 12–14°C, with no significant changes. This period coincided with a sharp diversification of Siberian biota, resulting in a two to threefold increase in diversity (Figure 11). Global paleotemperature assumptions propose a substantial increase from ~12°C in the Kungurian to 25°C in the Changhsingian at the end of the Early Permian and during the rest of the Permian (Figure 10b) (Scotese et al., 2021). The paleomagnetic and provenance study suggest that the Siberian platform during the Permian migrated northwards to the north pole (Figure 12a), whereas the obtained biotic paleotemperature data suggests the position of the northern margin of the platform at approximately 60–65° N (Figure 12b). However, thick coals continued to form on the platform during entire Permian and Middle to Late Triassic, suggesting that they remained below the Arctic Circle at 66.5° N under modern interglacial conditions (Kopansky et al., 2022).

Siberian Platform is a huge landmass that during Devonian‐Triassic stretched within several climatic zones (Boucot et al., 2013b). Our study summarizes the distribution of paleotemperatures within the Siberian Platform for each chronostratigraphic stage (Figure 10). The data from the Devonian‐Mississippian and the earliest Pennsylvanian (Bashkirian) show a general latitudinal gradient shift. However, the middle Pennsylvanian‐Permian–Triassic records reveal unusual tendencies and unexpected temperatures at different latitudes, inconsistent with the general latitudinal gradient model (Pianka, 1966). This suggests potential inaccuracy in the temperature calculations. The temperature variation in the post‐early Pennsylvanian period is not significant, usually not exceeding 3–5°C, but the general paleotemperature pattern clear (Figure 10). It should be noted that the Siberian Platform drifted northward, rotated counterclockwise, and stretched latitudinally during the post‐Devonian time, contrasting with its more meridionally elongated shape in the pre‐Pennsylvanian period (Figure 12). Further development and improvement of the biotic paleotemperature method could enable its use in regional and subregional paleoclimatic reconstructions.

Recent climate changes and Permian climate models indicate significant climate shifts during global warming and glacial episodes at high latitudes (McGlone et al., 2010; Penn et al., 2018). The Siberian Platform during the Late Paleozoic, occurred within subtropics, mid to high latitudes (Figure 12), potentially exhibiting more contrasting climate variations than other regions. The global temperature change model (Scotese et al., 2021) suggests a warm global climate during the middle Devonian, followed by a progressive temperature decline toward the earliest Pennsylvanian, consistent with the proposed here paleoclimate model of the Siberian Platform (Figure 10). The Moscovian‐Wuchiapingian climate in Siberia was persistently cool‐cold, with annual temperature variations of 7–10°C, whereas Scotese et al. (2021) suggest progressive global warming from 12°C during the Asselian‐Sakmarian transition to 25°C in the Changhsingian (Figure 10b). This strongly contradicts the Siberian records, where climate change should be even more pronounced. The Triassic fauna in Siberia, although more diverse than the Permian fauna, does not show a significant shift toward regional warming (Figure 10a). Further improvement and development of the biotic paleotemperature recognition method may resolve this discrepancy.

It is acknowledged that not all proposed biotic paleotemperature indices accurately depict real temperatures in different regions. We anticipate that the involvement of paleontologists studying various taxonomic groups will help refine these indices. Over time, this database is envisioned to evolve into a valuable tool for constructing and refining paleobiogeographic, paleogeographic, and paleotectonic maps and models. Furthermore, the proposed database and tools can serve as a reference and verification resource for the broader geological community. If our work garners interest and support from geologists, we plan to expand both the range of faunal groups and associated age ranges. The impact of taphonomic bias in our study is minimal as we focused on restricted groups of fossils, specifically shelly fossils such as ammonoids, bivalves, brachiopods, nautiloids, and ostracods. These fossils share similar preservation modes and durability, reducing the potential for significant taphonomic variations. It should be noted that the paleotemperature data discussed in this paper rely on the presence or absence of taxa and are largely independent of sampling size and intensity, making the paleotemperature estimates robust against taphonomic bias. However, it is crucial to acknowledge that taphonomic proxies may become a more prominent concern when dealing with diverse fossils exhibiting variable preservation modes, especially in terrestrial settings.

## 8 CONCLUSION

This article introduces a novel methodology for estimating paleotemperatures using paleontological taxa spanning from generic to family levels in diverse paleoecological settings. Although in the early stages of establishing a robust foundation for biotic paleotemperature estimation, our ultimate objective is to craft a versatile tool capable of addressing various geological challenges. The application of the developed algorithm, particularly in calculating the average temperature of sea‐surface water based on all fossils within each time slice, has proven to be reliable and productive, as evidenced in the Late Paleozoic basins of Siberia. Crucially, the approach outlined for the database and tools is not restricted to specific stratigraphic intervals or taxonomic groups. Should this research capture the interest of paleontologists investigating a broad spectrum of taxonomic groups, the proposed database has the potential to emerge as a dependable tool for both paleontologists and geologists specializing in various fields. As we refine the paleotemperature estimates from taxonomy and expand the database to encompass diverse regions, a multitude of geological challenges, including those in paleoclimatology, paleogeography, paleooceanology, paleobiogeography, paleotectonics, and potentially others, can be effectively addressed. The method proposed in this article significantly complements the existing methods of surface seawater. The initial data better be obtained from the literature that includes information on the paleoenvironments, bathymetry, paleo‐coordinates and reliable chronostratigraphy. The best results on the initial evaluation of the sea‐surface paleotemperature (shallow‐water setting).

## AUTHOR CONTRIBUTIONS

**Vladimir I. Davydov:** Conceptualization (equal); data curation (equal); formal analysis (equal); investigation (equal); methodology (equal); project administration (equal); resources (equal); supervision (equal); validation (equal); writing – original draft (equal). **Eugeny V. Karasev:** Conceptualization (equal); data curation (equal); formal analysis (equal); investigation (equal); methodology (equal); resources (equal); software (equal); validation (equal); visualization (equal); writing – review and editing (equal). **Elizaveta V. Popova:** Data curation (equal); investigation (equal); resources (equal); validation (equal). **Vladislav I. Poletaev:** Investigation (equal); resources (equal); validation (equal).

## CONFLICT OF INTEREST STATEMENT

The authors declare no conflicts of interest relevant to this study.

## Supporting information


Appendix S1.

## Data Availability

The PaleoSib relational database consists of seven tables with raw data that are uploaded in CSV format to the Zenodo repository (https://doi.org/10.5281/zenodo.13227985) and can be accessed using open API at: (https://biogeolog.paleobotany.ru/paleosib/) provided by Directus ver. 8 (https://github.com/directus/v8‐archive). The Zenodo repository also contains several tables that contain prepared data for the PBTV Shiny Application. The source code of the PBTV Shiny Application is available under a CC BY 4.0 license on GitHub (https://github.com/mironcat/pbtv).
