# Supplementary material for: Method of estimating sea‐surface paleotemperatures through biotic proxies: A case study in Upper Paleozoic paleoclimatic, paleogeographic and paleotectonic reconstructions of Siberia
Source: Ecol Evol. 2024 Nov 7;14(11):e70265. doi: 10.1002/ece3.70265 (PMC11542995; doi:10.1002/ece3.70265)
Supplement: Supplementary file 1 — Appendix S1. [file ECE3-14-e70265-s001.docx]

**The literature from where the data for the PaleoSib DB were compiled**

Abramov, B. C.; Grigorieva, A. D. Biostratigraphiya i Brakhiopody Permi Verkhoyaniya [Biostratigraphy and Brachiopoda from the Permian of Verchoyan]. 1988. Nauka, Moscow: 208 pp, 32 plates.

Afanasyeva G.A. New chonetids (brachiopods) from the Permian deposits of Verkhoyan // Paleontological Journal. 1988. V. 22 (2) P. 108–112.

Afitskiy A.I. Pervaya nakhodka Rhabdoceras na severo-vostoka SSSR // Paleontologicheskii Zhurnal. 1965. V. 1965 (3) P. 137–138.

Andrianov, V. N., Korostelev V. I. Permskie i nekotorye kamennougol'nye ammonoidei severo-vostoka Azii. Novosibirsk: Nauka, 1985.

Aristov D.S. New and little known Grylloblattida (Insecta) from intertrappean deposits of the Tunguska Basin of Siberia // Paleontological Journal. 2011. V. 45 (5) P. 537–545.

Aristov D.S. New and little-known Eoblattida (Insecta) from the Paleozoic of Russia // Paleontological Journal. 2013. V. 47 P. 272–282.

Aristov D.S. New Grylloblattida (Insecta) from the Middle and Upper Permian of Russia // Far Eastern Entomologist. 2008. V. 188 P. 1–7.

Aristov D.S. Revision of the Family Tomiidae (Insecta: Grylloblattida) // Paleontological Journal. 2003. V. 37 P. 31–38.

Aristov D.S. The fauna of grylloblattid insects (Grylloblattida) from the end of the Late Permian to the first half of the Triassic // Paleontological Journal. 2004. V. 38 P. 514–521.

Arkadiev V.V., Vavilov M.N. Anisian-Ladinian Boundary in Boreal Region Based on Ammonoidea // Palaeontographica Abteilung A. 1989. V. 207 P. 49–78.

Arkadiev V.V., Vavilov M.N. Middle Triassic Parapopanoceratidae and Nathorstitidae (Ammonoidea) of Boreal Region: Internal Structure, Ontogeny and Phylogenetic Patterns // Geobios. 1984. V. 17 (4) P. 397–415.

Astafieva M.M. On the representatives of the genus Maitaia (Bivalvia) // Paleontological Journal. 1989. V. 23 (3) P. 11–19.

Astafieva M.M. The first occurrence of members of the genus Trabeculatia (Bivalvia) in the Permian deposits of the northeastern USSR // Paleontological Journal. 1987. V. 20 (2) P. 19–24.

Astafieva, M. M. (1993). Permian Inoceramus-like bivalves of Russia. Trudy Paleontol. Inst. Ross. Akad. Nauk, 246, 1-128. (In Russian)

Astafieva-Urbajtis K.A. The genus Myonia (Bivalvia) in the Permian of the northeast of the USSR // Paleontological Journal. 1976. V. 10 (1) P. 23–36.

Astafieva-Urbajtis K.A. Vacunella Waterhouse and Cunavella gen. nov. (Bivalvia) from the upper Paleozoic of the USSR // Paleontological Journal. 1990. V. 24 (2) P. 1–9.

Astaf'yeva M.M. Bivalved mollusks of the genus Kolymia // Paleontological Journal. 1988. V. 22 (1) P. 17–26.

Astaf'yeva M.M. Cigarella - a new genus of inoceramid-like bivalve molluscs // Paleontological Journal. 1988. V. 22 (4) P. 17–24.

Astaf'yeva M.M. The Permian bivalved molluscs Parainoceramus and Kolymia // Paleontological Journal. 1987. V. 20 (4) P. 23–31.

Astaf'yeva-Urbaytis K.A. The genus Exochorhynchus from the upper Paleozoic deposits in the USSR and Mongolia // Paleontological Journal. 1981. V. 15 P. 29–36.

Baranov V.V. New Devonian Brachiopods from Northeastern Russia // Paleontological Journal. 2007. V. 41 (3) P. 252–259.

Baranov V.V. The Middle and Upper Devonian in southeastern flank of the Siberian Platform (southern Verkhoyansk region, Sette-Daban Mountain Range) // Stratigraphy and Geological Correlation. 2007. V. 15 (5) P. 470–484.

Becker-Migdisova E.E. Iskopaemye nasekomye iz Triasa Sibiri // Doklady Akademii Nauk SSSR. 1955. V. 105 P. 1100–1103.

Belskaya T.N. Organogennyye postroyki v devone Sayano-Altayskoy gornoy oblasti [Organic structures in the Devonian of the Altai-Sayan mountainous regions]. 1975. 100-107 p.

Biakov A.S. Complete succession of the Permian in northeast Asia: paleontological evidence form the presence of Changhsingian analogues // Doklady Earth Sciences. 2001. V. 378 P. 399–401.

Biakov A.S. New Inoceramus-like bivalves of the genus Kolymia Licharew from the Middle Permian of northeast Asia // Paleontological Journal. 2012. V. 46 P. 552–559.

Biakov A.S. New Inoceramus-like bivalves of the genus Praekolymia Biakov from the Lower Permian of the western Verkhoyansk region, northeastern Asia // Paleontological Journal. 2014. V. 48 P. 571–574.

Biakov A.S. New Permian Pteronites (Bivalvia, Pinnidae) from the Lower Permian of the Omolon Massif, northeastern Asia // Paleontological Journal. 2013. V. 47 P. 363–365.

Biakov A.S. New records of bipolar nuculanid bivalves of the genus Glyptoleda in the Permian of northeastern Asia // Paleontological Journal. 2016. V. 50 P. 573–577.

Biakov A.S. New species of astartids and pholadomyids (Bivalvia) and beds with fauna from the Lower Permian of the Omolon Massif, northeastern Asia // Paleontological Journal. 2005. V. 39 (2) P. 133–140.

Biakov A.S. New species of Inoceramus-like bivalves of the genus Aphanaia Koninck from the Lower Permian of northeast Asia // Paleontological Journal. 2011. V. 45 P. 5–12.

Biakov A.S. New species of Inoceramus-like bivalves of the subfamily Kolymiinae from the Middle Permian of northeastern Asia // Paleontological Journal. 2018. V. 52 P. 1–8.

Biakov A.S. New species of Permian inoceramid bivalves from northeast Russia // Paleontological Journal. 1992. V. 26 (1) P. 32–46.

Biakov A.S. New species of the Inoceramus-like bivalve genus Maitaia Marwick from the Permian of northeastern Asia // Paleontological Journal. 2013. V. 47 P. 463–469.

Biakov A.S. Permian biostratigraphy of the northern Okhotsk region (northeast Asia) // Stratigraphy and Geological Correlation. 2007. V. 15 (2) P. 161–184.

Biakov A.S. Permian ctenodont bivalves from northeastern Russia // Paleontological Journal. 1998. V. 32 (2) P. 129–132.

Biakov A.S. Permian Inoceramus-like mollusks of the genus Intomodesma Popov // Paleontological Journal. 1991. V. 25 (4) P. 159–164.

Biakov A.S. Two new zonal species of inoceram-like bivalves from the Upper Permian of northeastern Asia // Paleontological Journal. 1999. V. 33 P. 229–231.

Biakov A.S., Kutygin R.V. A new most ancient Permian Inoceramus-like bivalve of the genus Aphanaia Koninck from northeastern Asia // Paleontological Journal. 2015. V. 49 P. 356–360.

Biakov, A. S., 2008. New ideas on the system of the Permian Inoceramus-like bivalves of the eastern Boreal Zone//Paleontological Journal.

Bogoslovsky B.I. Devonskie ammonoidei I. Agoniatitidy // Akademiya Nauk SSSR, Trudy Paleontologicheskogo Instituta. 1969. V. 124 P. 1–328.

Budnikov I.V., Sivchikov V.E., Durante M.V., Betekhtina O.A., Kletz A.G. (1998). The scheme of the Upper Paleozoic. - 85 p.

Bychkov Y.M. Novye pozdnetriasovye trakhitseratidy severo-vostoka SSSR // Kolyma. 1973. V. 1973 (10) P. 35–38.

Bychkov Y.M. Pervye Tibetitidy na Severo-Vostoke SSSR // Kolyma. 1974. V. 1974 (8) P. 42–43.

Dagis A.S. K sisteme i filogenii longobarditid // Akademiya Nauk SSSR, Sibirskoe Otdelenie, Institut Geologii i Geofiziki. 1987. V. 688 P. 63–70.

Dagis A.S. Triassic brachiopods of Siberia. Moscow: Nauka Publishing House. 1965. 186 p.

Dagis A.S., Dagis A.A., Ermakova S.P. Triasovaya Fauna Severo-Vostoka Azii. 1996. 1-232 p.

Dagis A.S., Egorov A.Y., Kazakov A.M. et al. Stratigrafiya Triasovykh otlozheniy yugo-vostochnogo Tajmyria // Verkhniy Paleozoy i Trias Sibiri. Akademiya Nauk SSSR, Sibirskoe Otdelenie Trudy Instituta Geologii I Geofiziki. 1989. V. 732 P. 71–91.

Dagis A.S., Ermakova S.P. Boreal'nye Pozdneolenekskie Ammonoidei // Trudy Instituta Geologii i Geofiziki. 1988. V. 714 P. 1–135.

Dagis A.S., Ermakova S.P. Early Olenekian ammonoids of Siberia // Trudy Akademiya Nauk SSSR. 1990. V. 737 P. 1–112.

Dagys A. A new late Olenekian (Triassic) ammonoid of low palaeolatitude affinity from Arctic Asia (Eastern Taimyr) // Palaeontologische Zeitschrift. 1997. V. 71 P. 217–220.

Dagys A., Ermakov S. Induan (Triassic) ammonoids from north-east Asia // Revue de Paleobiologie. 1996. V. 15 (2) P. 401–447.

Dagys A.S. Triassic Brachiopods (Morphology, Classification, Phylogeny, Stratigraphical Significance and Biogeography). 1974. 1-387 p.

Dagys A.S., Kurushin N.I. Triasovye brakhiopody i dvustvorchatye mollyuski severa sredney Sibiri // Akademiya Nauk SSSR, Sibirskoe Otdelenie Trudy Instituta Geologii I Geofiziki. 1985. V. 633 P. 1–160.

Decision of the 5th interdepartmental regional stratigraphic meeting on Mesozoic deposits of the West Siberian Plain / editor Nesterov I.I. // Tyumen: ZapSibNIGNI, 1991, 54 p.

Decisions of the 2nd Interdepartmental Regional Stratigraphic Meeting on Precambrian and Phanerozoic of the North-East of the USSR (Magadan, March 10, 1975 and February 2-3, 1976) / Editorial board: N.A. Shilo, P.V. Babkin, Yu.M. Bychkov et al.. Magadan. 1978. 192 p. 16 table.

Decisions of the All-Union Meeting on the Development of Unified stratigraphic schemes of the Precambrian, Paleozoic and Quaternary system of Central Siberia, Part II (Middle and Upper Paleozoic), 1979 Edited by V. I. Krasnov. Leningrad, 1982, 130 p.

Decisions of the Third Interdepartmental Regional Stratigraphic Meeting on the Precambrian, Paleozoic and Mesozoic of the North-East of Russia / ed.: T.N. Koren, G.V. Kotlyar // St. Petersburg: ed. VSEGEI, 2009, 267 p.

Dobruskina I.A. Triassic Floras of Eurasia. 1982. 1-196 p.

Dubatolov V.N., Krasnov V.I. Paleobiogeography of the west Siberian sea in the Devonian // Russian Geology and Geophysics. 1993. V. 34 (4) P. 22–29.

Efremov J.A. First representative of Siberian early Tetrapoda // Comptes-Rendus des Seances de l'Academie des Sciences de l'URSS. 1939. V. 23 (1) P. 106–110.

Ermakova S.P. Sakhaitoides, a new Early Triassic ammonoid genus // Paleontological Journal. 1999. V. 33 P. 610–613.

Ermakova S.P. Systematic position of the genus Dieneroceras Spath (Ammonoidea, Ceratitida) // Paleontological Journal. 1974. V. 8 P. 128–131.

Ganelin V.G., Lazarev S.S. Revision of the Permian thin-ribbed Linoproductoids. Article 2. The "Cancrinella" ogonerensis Zavodowsky, 1960 Group // Paleontological Journal. 2000. V. 34 (1) P. 40–46.

Ganelin V.G., Lazarev S.S. Revision of the Permian thin-ribbed linoproductoids: 1. Linoproductoids of the "Cancrinella" koninckiana (Keyserling, 1846) group // Paleontological Journal. 1999. V. 33 (3) P. 243–253.

Grigorjeva A.D., Solomina R.V. A new genus of Licharewiinae (Brachiopoda) from the Permian of Verkhoyan'ye and the northeast USSR // Paleontological Journal. 1973. V. 7 (4) P. 473–477.

Gurari, F. G., Ed. (2004). Decision of the 6th Interdepartmental Stratigraphic Meeting on the consideration and adoption of refined stratigraphic schemes of the Mesozoic deposits of Western Siberia. Novosibirsk, SNIIGGIMS, 114. 31 schemes.

Ivanov A., Klets T., Lucas S.G. et al. Triassic marine fishes from Siberia, Russia // The Global Triassic. New Mexico Museum of Natural History and Science Bulletin. 2007. V. 41 P. 108–109.

K. V. Simakov, M. J. M. Bless, J. Bouckaert, R. Conil, M. H. Gagiev, Y. V. Kolesov, Y. I. Onoprienko, E. Poty, T. P. Razina, N. A. Shilo, L. V. Smirnova, M. Streel, and R. Swennen. 1983. Upper Famennian and Tournaisian deposits of the Omolon region (NE-USSR). Annales de la Societe Geologique de Belgique 106(2):335-399

Kashirtsev, A. S. "Polevoi atlas fauny permskikh otlozhenii Severo-Vostoka SSSR (Field Atlas of the Fauna from the Permian deposits of the Northeastern USSR). 1959. Moscow: Akad." Nauk SSSR.

Kazakov A. M. et al. Stratigraphy of Oil and Gas Basins of Siberia. Triassic System //Novosibirsk: Department “GEO”, Siberian Branch, Russian Academy of Sciences. – 2002. 322 p.

Kazakov A.M., Kurushin N.I. Stratigraphy of Norian and Rhaetian deposits in the northern Middle Siberia // Russian Geology and Geophysics. 1992. V. 33 (6) P. 1–8.

Khromykh V.G. Discovery of Lower Famennian Stromatoporoidea in the Kuznetsk Basin // Paleontological Journal. 2008. V. 42 (3) P. 227–231.

Klets A.G. Brachiopods of the family Elythidae from the Lower Permian of the Khabarovsk Territory // Paleontological Journal. 1987. V. 21 P. 31–36.

Klets A.G. New stratigraphically significant brachiopods from the late Paleozoic of southwestern Verkhoyan'ye // News on Paleontology and Stratigraphy. 1998. V. 1 P. 91–101.

Konstantinov A.G. A new ammonoid genus from the Carnian of the northern Okhotsk region // Paleontological Journal. 1999. V. 33 P. 132–135.

Konstantinov A.G. A revision of the early Carnian Trachyceratidae (Ammonoidea) of northeastern Asia // Paleontological Journal. 2012. V. 46 P. 453–460.

Konstantinov A.G. Arctophyllites, a new ammonoid genus from the Carnian of Northeast Asia // Paleontological Journal. 1995. V. 29 (3) P. 19–29.

Konstantinov A.G. Biostratigrafiya i ammonoidei verkhnego Aniziya severa Sibiri // Akademiya Nauk SSSR, Sibirskoe Otdelenie Trudy Instituta Geologii I Geofiziki. 1991. V. 787 P. 1–136.

Konstantinov A.G. Orientosirenites, a new ammonoid genus (Sirenitidae; Ammonoidea) from the upper Carnian of the Boreal Realm // Paleontological Journal. 2018. V. 52 P. 18–26.

Konstantinov A.G., Sobolev E.S., Yadrenkin A.V. Detailed biostratigraphy of Triassic deposits in the Lena lower reaches(northern Yakutia) // Russian Geology and Geophysics. 2007. V. 48 P. 721–736.

Krasnov V.I. Decisions of the interdepartmental meeting on the consideration and adoption of the regional stratigraphic chart of the Paleozoic formations of the West Siberian Plain. Novosibirsk: Sib. scientific research Institute of Geology, Geophysics and Mineral. resources // Novosibirsk: Sib. scientific research Institute of Geology, Geophysics and Mineral resources. 1999.

Krasnov V.I., Peregoedov L.G., Ratanov L.S., Fedoseev G.S. Regional stratigraphic chart of the Devonian Formations in the eastern part of the Altai-Sayan region // Geology and mineral resources of Siberia. 2018 P. 54–101.

Kurushin N.I. New taxa of pelagic and benthic bivalves from the Triassic of northeastern Russia // News on Paleontology and Stratigraphy. 1998. V. 1 P. 123–131.

Kurushin N.I. Triasovye Paleogeterodontnye i Geterodontnye Dvustvorki Sibiri [Triassic Paleoheterodont and Heterodont Bivalves of Siberia] // Trudy Instituta Geologii i Geofiziki. 1992. V. 742 P. 1–86.

Kurushin N.I., Truschelev A.M. Magnolobia: a new bivalve genus from the boreal Ladinian // Paleontological Journal. 2001. V. 35 (3) P. 243–248.

Kutygin R.V. On the Early Permian Somoholites (Ammonoidea) from the Verkhoyansk region // Paleontological Journal. 1999. V. 33 P. 516–521.

Kutygin R.V. Paratumaroceras, a new paragastrioceratid genus (Ammonoidea) from the Lower Permian of the western Verkhoyansk region // Paleontological Journal. 2003. V. 37 (3) P. 252–256.

Kutygin R.V. Spirolegoceratids (Ammonoidea) from northeastern Russia // Paleontological Journal. 1996. V. 30 (5) P. 506–514.

Kutygin R.V. Uraloceras subsimense, a new goniatite species from the Lower Permian of the Verkhoyansk region // News on Paleontology and Stratigraphy. 2004. V. 6-7 P. 125–131.

Kutygin R.V., Biakov A.S. Permian ammonoids of the Okhotsk region, northeast Asia // Paleontological Journal. 2015. V. 49 P. 1275–1281.

Kutygin R.V., Ganelin V.G. Permian ammonoids of the Kolyma-Omolon region: Kyrian association // Paleontological Journal. 2011. V. 45 (3) P. 249–259.

Kutygin R.V., Ganelin V.G. Permian ammonoids of the Kolyma–Omolon Region: Ogonerian Association // Paleontological Journal. 2013. V. 47 P. 1–10.

Kutygin R.V., Ganelin V.G., Biakov A.S. New records of the late Carboniferous ammonoid genus Eoshumardites in the Kolyma–Omolon Region, and notes on the evolution of Eoshumarditidae // Paleontological Journal. 2016. V. 50 P. 347–357.

Lazarev S.S. Brachiopods of the Tribe Horridoniini (Order Productida): 2. Evolution and Systematics // Paleontological Journal. 2005. V. 39 (2) P. 148–157.

Lazutkina O.F. Discovery of a bryozoan of the Paleozoic genus Batostomella in the Triassic // Paleontologiskii Zhurnal. 1963. V. 4 P. 126–128.

Leonova T.B., Kutygin R.V., Shilovsky O.P. New data on the composition and evolution of the Permian superfamily Popanocerataceae (Ammonoidea) // Paleontological Journal. 2005. V. 39 (5) P. 476–486.

Lutkevich E.M. Taimyria//Materialy po Paleontologii, Novye Semeystva i Rody. 1956. 71-72 p.

Martynov A.V. O neskolykikh nasekomykh iz kol'chuginskoy svity Kuznetskogo Basseyna // Izvestiya Akademii Nauk SSSR. 1935. V. 1935 P. 441–448.

Martynov A.V. On some new materials of Arthropoda from Kuznetsk-Basin // Izvestiya Akademii Nauk SSSR: Seriya Biologicheskaya. 1936. V. 6 P. 1251–1264.

Martynov A.V. Palaeozoic insects from the Kuznetzk Basin // Izvestiya glavnogo geologo-Razvedochnogo Upravleniya, Moskva. 1930. V. 49 (10) P. 1221–1248.

Martynova O.M. Novye nasekomye iz Permskikh i Mezozoiskikh otlozhenii SSSR [=New insects from the Permian and Mesozoic deposits of the USSR] // Materialy po Osnovam Paleontologii. 1958. V. 2 P. 69–94.

Martynova O.M. Sovremennye i vymershie verblyudki (Insecta, Raphidioptera) // Paleontologicheskii Zhurnal. 1961. V. 1961 (3) P. 73–83.

Mashchak M.S. Legend of the Anabar-Vilyui series of sheets of the State Geological Map of the Russian Federation, scale 1: 1,000,000 (third generation). Book 1. St. Petersburg. 1 p.

Milova L.V. Triassic-Jurassic stratigraphy and Bivalvia of northern Priokhotye // Akademia Nauk SSSR, Trudy Severo-Vost. Kompl. Nauchno. Issled. Institut. 1976. V. 65.

Molostovskaya I.I. Ostracodes from the Upper Permian Khivach Formation in Kolyma-Omolon Basin // Paleontological Journal. 2010. V. 44 (3) P. 282–286.

Morozova I.P. Pozdnepaleozoiskie mshanki severo-vostoka CCCP // Akademiya Nauk SSSR, Trudy Paleontologicheskogo Instituta. 1981. V. 188 P. 1–119.

Morozova I.P. Some Late Permian bryozoans from northeast USSR // Paleontological Journal. 1970. V. 4 P. 361–370.

Morozova I.P., Zharnikova N.K. On some new Triassic bryozoans // Paleontological Journal. 1984. V. 4 P. 72–79.

Muromtseva V.A., Guskov V.A. Permskie Morskie Otlozheniya I Dvustvorchatie Mollyuski Sovetskoi Arktiki. 1984. 1-154 p.

Nekhoroshev V.P. First occurence of Triassic bryozoans in the U.S.S.R // Akad. Nauk SSSR Doklady, nov. ser. 1949. V. 66 (3) P. 459–461.

Novokshonov V.G. Permian Scorpion Flies (Insecta, Panorpida) of the Families Kaltanidae, Permochoristidae and Robinjohnidae // Paleontological Journal. 1994. V. 28 (1) P. 79–95.

Polubotko I.V. Early Carnian Halobiidae of Northeast Asia // Paleontological Journal. 1980. V. 14 (1) P. 34–41.

Ponomarenko A.G. A New Beetle Species of the Genus Taldycupes (Taldycupedidae, Coleoptera) from the Permian of the Tunguska River Basin // Paleontological Journal. 2006. V. 40 (3) P. 295–296.

Ponomarenko A.G., Shcherbakov D.E. New lacewings (Neuroptera) from the terminal Permian and basal Triassic of Siberia // Paleontological Journal. 2004. V. 38 Suppl 2 P. 197–203.

Regional stratigraphic scheme of Devonian formations of the West Siberian oil and gas province / V.I. Krasnov, N.P. Kulkov, L.G. Peregoedov, etc.; editors: A.S. Efimov (Chief editor), etc. // Novosibirsk: SNIGGIMS, 2012, 42 p. (In Russian)

Rohdendorf B.B., Becker-Migdisova E.E., Martnova O.M. et al. Paleozoiskie nasekomye kuznetskogo basseina. 1961. 1-705 p.

Ruzhentsev V.E., Bogoslovskaya M.F. Namurskiy etap v evolyutsii ammonoidey. Rannenamyurskie ammonoidei // Akademiya Nauk SSSR, Trudy Paleontologicheskogo Instituta. 1971. V. 133 P. 1–382.

Rzhonsnitskaya M.A. Biostratigrafiya devona okrain Kuznetskogo basseyna, 1: Stratigrafiya [Devonian biostratigraphy of the Kuznetsk Basin margin, 1: Stratigraphy]. 1968. 1-287 p.

Rzhonsnitskaya M.A., Markovskii B.P., Yudina Y.A. et al. Late Frasnian Atrypida (Brachiopoda) from the South Urals, South Timan and Kuznetsk Basin (Russia) // Acta Palaeontologica Polonica. 1998. V. 43 (2) P. 305–344.

Sarycheva T.G. Brakhiopody verkhnego paleozoya Sibiri i Arktiki // Akademiya Nauk SSSR, Trudy Paleontologicheskogo Instituta. 1977. V. 162 P. 1–119.

Sarycheva T.G. Pozdnepaleozoyskie produktidy Sibiri i Arktiki [Upper Paleozoic productids of Siberia and the Arctic] // Akademiya Nauk SSSR, Trudy Paleontologicheskogo Instituta. 1977. V. 161 P. 1–220.

Sax V.N., Golbert A.V., Dagis A.S. and others. Decisions of the 3rd Interdepartmental Regional Stratigraphic Meeting on the Mesozoic and Cenozoic of Central Siberia, Novosibirsk, 1978. Novosibirsk.

Schastlivtseva N.P. Nekotoryye triasovyye ortotseratidy i nautilidy Severo-Vostoka SSSR. Some Triassic orthoceratids and nautilids from northeastern part of the USSR // Byulleten' Moskovskogo Obshchestva Ispytateley Prirody, Otdel Geologicheskiy. 1986. V. 61 (2) P. 122–129.

Schoch R., Milner A.R. Stereospondyli. 2000. 1-203 p.

Schram F.R. Miscellaneous late Paleozoic Malacostraca of the Soviet Union // Journal of Paleontology. 1980. V. 54 (3) P. 542–547.

Sharov A.G. Filogeniya ortopteroidnykh nasekomykh // Trudy Paleontologicheskogo Instituta Akademii Nauk SSSR. 1968. V. 118 P. 1–216.

Sharov A.G., Sinitshenkova N.D. New Palaeodictyoptera from the USSR // Paleontological Journal. 1977. V. 11 (1) P. 44–59.

Shimanskiy V.N. Kammenougol'nye Nautilida // Akademiya Nauk SSSR, Trudy Paleontologicheskogo Instituta. 1967. V. 115 P. 1–258.

Shimansky V.N. Upper Paleozoic Nautilida of the northern regions of the USSR // Bull. MOIP. 1990. T. 65 P. 60–74.

Shishkin M.A. Tungussogyrinus; a relict neotenic dissorophoid (Amphibia, Temnospondyli) from the Permo-Triassic of Siberia // Paleontological Journal. 1998. V. 32 (5) P. 521–531.

Sinitshenkova N.D. A new family of the Palaeodictyoptera from the Carboniferous of Siberia // Paleontological Journal. 1979. V. 13 P. 192–205.

Sinitshenkova N.D. A new insect family Aykhalidae from the upper Palaeozoic of Yakutia-Zakha (Insecta: Mischopterida = Megasecoptera) // Paleontological Journal. 1993. V. 27 1A P. 131–134.

Sinitshenkova N.D. Istopicheskoe razvitie vesiyanok // Akademiya Nauk SSSR, Trudy Paleontologicheskogo Instituta. 1987. V. 221 P. 1–142.

Sinitshenkova N.D. New mayflies (Insecta: Ephemerida = Ephemeroptera) from the intertrappean deposits of the Tunguska Basin, Siberia // Paleontological Journal. 2013. V. 47 P. 84–88.

Sobolev E.S. Novye vidy sveriutykh Nautiloidey iz srednego i verkhnego Triasa Sibiri [New species of coiled nautiliods (Mollusca) from the Middle and Upper Triassic of Siberia] // News on Paleontology and Stratigraphy. 1998. V. 1 P. 133–151.

Sobolev E.S. Triasovye nautilidy severo-vostochnoy Azii // Akademiya Nauk SSSR, Sibirskoe Otdelenie Trudy Instituta Geologii I Geofiziki. 1989. V. 727 P. 1–193.

Sobolev E.S., Budnikov I.V., Klets A.G. et al. Late Bashkirian Amminoids and Nautiloids from Western Verkhoyansk Region // Paleontological Journal. 1998. V. 32 (5) P. 13–25.

Sobolev Y.S. A revision of the Triassic genus Phaedrysmocheilus (Nautiloidea) // Paleontological Journal. 1985. V. 19 P. 49–56.

Solomina R.V. A new upper Permian brachiopod genus from the northeastern USSR // Paleontological Journal. 1985. V. 19 (2) P. 117–121.

Solomina R.V. New Permian brachiopods from Verkhoyan // Paleontological Journal. 1988. V. 22 (1) P. 39–50.

Storozhenko S.Y., Vršanský P. New fossil family of the order Grylloblattida (Insecta: Plecopteroidea) from Asia // Far Eastern Entomologist. 1995. V. 19 P. 1–4.

Sumrall C.D. First definite record of Permian edrioasteroids: Neoisorophusella maslennikovi n sp from the Kungurian of northeast Russia // Journal of Paleontology. 2009. V. 83 (6) P. 990–993.

Tchudinova I.I. New data on the Lower Permian tabulate corals // Paleontological Journal. 2000. V. 34 (3) P. 252–257.

Turbin M.T. Decisions of the Fourth Interdepartmental Regional Stratigraphic Meeting on the Precambrian and Phanerozoic of the South of the Far East and Eastern Transbaikalia (Khabarovsk, 1990). Khabarovsk: KHGGGP. 1994. 124 p.

Ustritsky V.I., Chernyak G.E. Biostratigrafiya i Brakhiopody Verknego Paleozoya Taimyra [Biostratigraphy and Brachiopods of the upper Paleozoic of Taimyr] // Trudy Nauchno-Issledoratel'skogo Instituta Geologii Arktiki. 1963. V. 134 P. 1–139.

Vavilov M.N. Ontogenetic development of Anisian ammonoids of Taymyr // Paleontological Journal. 1969. V. 3 P. 184–190.

Vavilov M.N. Ontogenetic development of Early Triassic ceratites of the genus Koninckites // Paleontological Journal. 1969. V. 3 P. 120–123.

Vavilov M.N. Some Anisian ammonoids of northern Siberia // Paleontological Journal. 1978. V. 12 P. 331–343.

Vavilov M.N., Arkadiev V.V. Ammonoid remains in the body chambers of the Late Carnian ammonoid Proarcestes from Kotel'nyi Island (New Siberian Islands) // Paleontological Journal. 1998. V. 32 (2) P. 34–39.

Vavilov M.N., Arkadiev V.V. Novye i redkie Ammonoidei srednego i pozdnego Triasa sredney Sibiri // Akademiya Nauk SSSR, Sibirskoe Otlodenie, Trudy Instituta Geologii i Geofiziki. 1986. V. 648 P. 38–47.

Vavilov M.N., Zakharov Y.D. Reviziya Rannetriasovogo roda Pachyproptychites. 1976. 60-67 p.

Vishnyakova V.N. New Paleozoic Spiloblattinidae from Russia // Paleontological Journal. 1993. V. 27 1A P. 135–147.

Yegorov A.N., Popov L.E. A new Lower Permian lingulid from the Siberian Platform // Paleontological Journal. 1990. V. 24 (4) P. 107–112.

Yolkin E.A., Gratsianova R.T., Bakharev N.K. et al. Facies and faunal associations of the Telengitian (Emsian) in its type locality. Calgary: Canadian Society of Petroleum Geologists. 1988. 193-207 p.

Zakharov Y.D. Ammonoid succession of Setorym River (Verkhoyansk area) and problem of Permian-Triassic boundary in Boreal Realm // Journal of China University of Geosciences. 2002. V. 13 P. 107–123.

Zakharov Y.D. Examples of Late Olenekian invertebrate successions: Paper 2. Arctic Siberia (Mengilyakh Creek) // Albertiana. 2007. V. 35 P. 52–58.

Zakharov Y.D. Novaya nakhodka Pearylandites v Triase vostoka SSSR. 1976. 68-72 p.

Zakharov Y.D. Rannetriasovye ammonoidei vostoka SSSR. 1978. 1-224 p.

Zakharov Yu.D., Biakov A.S., Horacek M., Kutygin R.V., Sobolev E.S., Bond D.P.G. (2020). in Siberia (Verkhoyansk Region) and Neighbouring Areas During Permian–Triassic Large Igneous Province Activity // In: Metadata of the chapter that will be visualized online / Springer Nature Switzerland AG, Chapter 10.

Zalessky G. Sur deux restes d'insectes fossiles provenant du bassin de Kousnetzk et sur l'age géologique des dépôts qui les renferment // Bulletin de la Société Géologique de France, Cinquième Série. 1935. V. 5 P. 687–695.

Zalessky M.D. Observations sur les insectes trouvés dans les dépôts a charbon du bassin de Kousnetzk et sur l'age de ces derniers d'après la fauna entomologique // Bulletin de la Société Géologique de France, Cinquième Série. 1931. V. 1 P. 209–218.

Zavodowsky V.M. Chivatschella // Novye Vidy Drevnikh Rasteniy i Bespozvonochnykh SSSR. 1968. 125-126 p.

Zavodowsky V.M. Delthyriacea //Novye Vidy Drevnikh Rasteniy i Bespozvonochnykh SSSR. 1968. 169-173 p.

Zavodowsky V.M. Dielasmatidae //Novye Vidy Drevnikh Rasteniy i Bespozvonochnykh SSSR. 1968. 180-181 p.

Zavodowsky V.M. Echinoconchidae, Linoproductidae, Productidae //Novye Vidy Drevnikh Rasteniy i Bespozvonochnykh SSSR. 1968. 92-97 p.

Zavodowsky V.M. Novyy Permskiy Predstavitel' Konokardiid Severo-Vostoka SSSR [New Permian representives of the Conocardiidae from northeastern SSSR]. 1960. 31-33 p.

Zavodowsky V.M. Orthotetacea // Novye Vidy Drevnikh Rasteniy i Bespozvonochnykh SSSR. 1968. V. 2 P. 89–90.

Zavodowsky V.M. Scyphozoa //Novye Vidy Drevnikh Rasteniy i Bespozvonochnykh SSSR. 1968. 45-46 p.

Zavodowsky V.M. Spiriferacea //Novye Vidy Drevnikh Rasteniy i Bespozvonochnykh SSSR. 1968. 149-160 p.
